# Klotho levels and biological age acceleration: Insights from a diverse cohort of middle-aged and elderly individuals

**DOI:** 10.1371/journal.pone.0343429

**Published:** 2026-03-02

**Authors:** Mengna Huang, Guoxin Huang, Chaoshen Wu, Lei Zhu, Bin Pei, Hongwei Wang, Da Qian, Puzhao Wu

**Affiliations:** 1 Research Management Center, Changshu Hospital Affiliated to Soochow University, Changshu No.1 People’s Hospital, Changshu, China; 2 Department of Evidence-Based Medicine Center, Xiangyang No.1 People’s Hospital, Hubei University of Medicine, Xiangyang, China; 3 Central Laboratory, Changshu Hospital Affiliated to Soochow University, Changshu No.1 People’s Hospital, Changshu, China; 4 Department of Clinical Laboratory, Xiangyang No.1 People’s Hospital, Hubei University of Medicine, Xiangyang, China; 5 Department of Burn and Plastic Surgery-Hand Surgery, Changshu Hospital Affiliated to Soochow University, Changshu No.1 People’s Hospital, Changshu, China; 6 Department of Vascular Surgery/Interventional Medicine, Xiangyang No.1 People’s Hospital, Hubei University of Medicine, Xiangyang, Hubei Province, China; Sarich Neuroscience Research Institute, AUSTRALIA

## Abstract

**Objective:**

Aging is characterized by progressive physiological and psychological changes, leading to decreased cellular metabolism and increased vulnerability to age-related diseases.

**Methods.:**

In this study, we examined the relationship between circulating Klotho levels and biological age acceleration (BAA) in a representative cohort of middle-aged and older adults. Data were obtained from the National Health and Nutrition Examination Survey (NHANES, 2007–2010), including 5,654 participants aged 45–85 years. Serum Klotho concentrations were quantified using ELISA, while biological age was estimated with the BioAge R package.

**Result:**

Linear regression analyses demonstrated a robust inverse association between log-transformed Klotho levels and BAA across all statistical models, with reductions of −1.06 (95% CI: −1.77 to −0.36, p = 0.005), −1.44 (95% CI: −2.15 to −0.73, p < 0.001), and −1.30 (95% CI: −2.20 to −0.40, p = 0.01). Consistent results were observed in logistic regression models, where higher Klotho concentrations were linked to lower odds of accelerated aging (OR = 0.72, 95% CI: 0.59–0.88, p = 0.002; OR = 0.63, 95% CI: 0.51–0.77, p < 0.0001; OR = 0.62, 95% CI: 0.46–0.84, p = 0.01). Subgroup analyses revealed significant associations in women, participants over 60 years of age, and individuals without chronic illnesses. Interaction analyses further indicated that age (p-interaction = 0.002), alcohol intake (p-interaction = 0.04), and diabetes status (p-interaction = 0.03) significantly modified the Klotho–BAA relationship. Moreover, restricted cubic spline analysis showed a non-linear L-shaped dose-response pattern, suggesting that the protective effect of Klotho becomes more pronounced above a certain threshold.

**Conclusion:**

Collectively, these findings underscore the pivotal role of Klotho in the biology of aging and emphasize the importance of accounting for demographic and health-related modifiers in future investigations.

## 1 Introduction

Aging is an unavoidable and complex biological process experienced by all individuals, characterized by a progressive decline in both physiological and psychological functions. With advancing age, the body undergoes widespread cellular and systemic alterations, including reduced metabolic activity, deterioration of organ function, and diminished self-regulatory and regenerative capacity. These changes drive structural and functional modifications across tissues and organs, thereby increasing vulnerability to a broad spectrum of age-related conditions, including cardiovascular disease, diabetes, osteoporosis, and neurodegenerative disorders. Collectively, such alterations contribute to higher mortality risk [1]. Beyond individual health outcomes, aging also imposes profound social and economic challenges, placing a growing strain on healthcare systems and societies globally [2].

The Klotho gene plays a pivotal role in the biological mechanisms underlying aging and is widely recognized for its anti-aging functions. First described by Kuro-o et al. (1997) using a mouse knockout model, its importance was highlighted through striking phenotypic consequences. Mice deficient in Klotho displayed features reminiscent of accelerated human aging, including growth retardation, reduced lifespan, hypokinesia, gait disturbances, reproductive and thymic atrophy, atherosclerosis, ectopic calcification, osteoporosis, skin atrophy, impaired maturation of gonadal cells, emphysema, and abnormalities of the pituitary gland [3]. These observations established Klotho as a key regulator of physiological homeostasis and a central determinant in the modulation of aging processes.

In recent decades, research on the biological mechanisms of aging has expanded considerably, with the Klotho gene and its protein product becoming a central focus in gerontology. A growing body of evidence underscores its role as both a biomarker of aging and a protective factor against age-related diseases. For instance, Shahzamani et al. (2024) reported a strong association between reduced serum Klotho levels and greater severity of COVID-19 in older adults, implicating Klotho in the regulation of inflammatory responses [4]. Similarly, Yang et al. (2024) showed that low serum α-Klotho serves as a robust predictor of all-cause and cardiovascular mortality in a large population-based cohort, underscoring its clinical significance in age-associated conditions such as hypertension and diabetes [5]. Similarly, Wang et al. (2024), using NHANES data from adults aged 40–69, demonstrated that higher serum α-Klotho levels were linked to lower hearing thresholds and a reduced risk of hearing loss, suggesting a protective influence on auditory health in aging populations [6]. Complementing these findings, Abraham and Li (2022) provided a comprehensive review summarizing Klotho’s diverse biological functions, highlighting its anti-aging properties and therapeutic potential [7]. Collectively, these studies reinforce the pivotal role of Klotho in aging research and point to its promise as a target for interventions aimed at improving health outcomes across the lifespan.

Although considerable research has explored the link between Klotho and aging, its relationship with biological age acceleration (BAA) remains insufficiently understood. BAA serves as an individualized measure of aging, reflecting the gap between a person’s biological age derived from a composite of biomarkers and their chronological age. Unlike chronological age, which marks the passage of time, BAA provides a more precise evaluation of physiological and biological status, offering deeper insight into an individual’s actual aging trajectory. Clarifying the connection between Klotho and BAA may reveal new aspects of the molecular mechanisms that drive aging and identify therapeutic opportunities to foster healthy aging and reduce the risk of age-associated diseases.

To address this gap, the present study investigates the association between serum Klotho concentrations and BAA among middle-aged and older adults. Using data from the National Health and Nutrition Examination Survey (NHANES, 2007–2010), we evaluated whether Klotho exerts protective effects against biological aging and assessed the influence of demographic and health-related factors on this relationship. By doing so, this study provides important insights into Klotho’s role in the aging process. The findings may contribute to the development of novel approaches for preventing and managing age-related disorders, thereby improving quality of life and alleviating the growing healthcare and societal burden of aging.

## 2 Materials and methods

### 2.1 Data source

**Fig 1:**
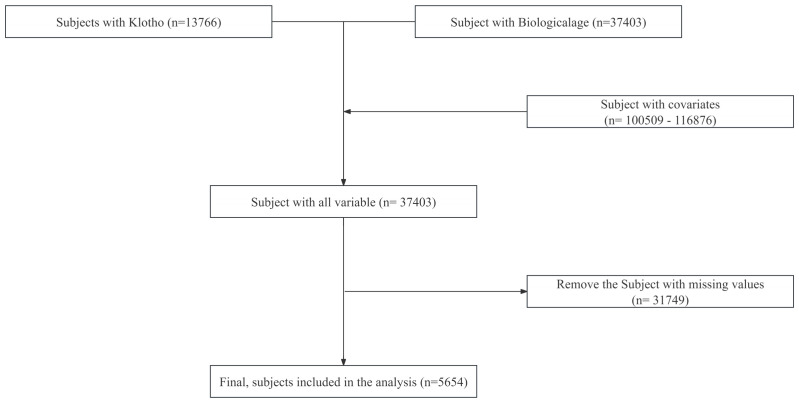
Flowchart showing the participants’ enrollment procedure.

This study used data from the NHANES collected during the 2007–2010 cycles. As this study used publicly available anonymized data from the NHANES website for secondary analysis, ethical approval and participant consent were not required. A total of 116,876 individuals were initially considered. Following the integration of relevant variables and the exclusion of participants with incomplete data, the final analytic cohort comprised 5,654 individuals. Fig 1 provides an overview of participant characteristics and outlines the data cleaning process. Since the analysis was based on publicly available, de-identified NHANES data, separate ethical approval and informed consent were not required. Meanwhile, the data from this study have been uploaded to the Figshare website, with the DOI number 10.6084/m9.figshare.29998939.

### 2.2 Klotho serum measurement

Serum Klotho concentrations were measured using an enzyme-linked immunosorbent assay (ELISA) performed on fresh-frozen blood samples stored at –80 °C. Quantification was carried out with a commercially available kit (IBL International, Japan). Each sample was analyzed in duplicate, and the mean value was used as the final Klotho concentration (pg/mL). Samples with duplicate measurements differing by more than 10% were flagged and reanalyzed to ensure accuracy. The assay demonstrated a sensitivity of 6 pg/mL [8].

### 2.3 Measurement of biological age

Biological age was estimated using the BioAge R package developed by Dayoon Kwon and Daniel W. Belsky, available on GitHub (https://github.com/dayoonkwon/BioAge) [9]. This tool applies a set of validated biomarkers to generate a quantitative measure of biological aging. The calculation incorporated the following biomarkers: natural log-transformed C-reactive protein (Ln-CRP), serum creatinine, glycated hemoglobin (HbA1c), serum albumin, total cholesterol, blood urea nitrogen, alkaline phosphatase, and systolic blood pressure. BAA was defined as the difference between biological age and chronological age. A positive BAA value indicates that biological age exceeds chronological age, reflecting an elevated risk for age-related conditions. In comparison, a negative value suggests slower biological aging and potentially lower disease susceptibility [10].

The biomarkers used to estimate biological age, including Ln-CRP (C-reactive protein), serum creatinine, HbA1c, serum albumin, total cholesterol, blood urea nitrogen, alkaline phosphatase, and systolic blood pressure, were selected for their well-established associations with aging processes and age-related diseases. Collectively, they capture the functional integrity of multiple physiological systems that contribute to aging.

- *Ln-CRP:* A marker of systemic inflammation, linked to aging and conditions such as cardiovascular disease, diabetes, and neurodegeneration.
- *Serum creatinine:* An indicator of kidney function, where elevated levels often reflect renal impairment, a key driver of accelerated aging and morbidity.
- *HbA1c:* A measure of long-term glycemic control, with higher values associated with diabetes risk and age-related complications.
- *Serum albumin:* A marker of nutritional status and liver function, both essential for homeostasis and known to decline with age.
- *Total cholesterol:* Strongly associated with cardiovascular disease, which is highly prevalent in aging populations.
- *Blood urea nitrogen:* Another marker of renal function, where elevated concentrations indicate dysfunction that contributes to age-related decline.
- *Alkaline phosphatase:* Reflects bone turnover and liver function, both of which undergo age-related changes.
- *Systolic blood pressure:* A key determinant of cardiovascular health, with higher values commonly observed with aging and linked to increased cardiovascular risk.

These biomarkers were chosen for their ability to represent the multifaceted nature of aging across diverse organ systems. By reflecting systemic inflammation, metabolic regulation, cardiovascular integrity, renal and hepatic function, as well as nutritional and bone health, they provide a comprehensive measure of biological aging that extends beyond simple chronological age.

### 2.4 Covariates

To minimize the influence of potential confounders, fifteen covariates encompassing demographic, lifestyle, socioeconomic, and health-related factors were included in the analysis. Demographic variables consisted of sex, age, race/ethnicity, marital status, and educational attainment. Lifestyle factors included body mass index (BMI), alcohol consumption, smoking status, physical activity, and serum vitamin D levels. Socioeconomic status was assessed using the poverty level. Health-related covariates comprised the presence of cancer, hypertension, diabetes, and cerebrovascular disease.

BMI was calculated as weight (kg) divided by height squared (m²). Alcohol use was classified into five categories: never, former, mild, moderate, and heavy. Smoking status was categorized as never, former, or current smoker. Marital status was grouped as married, cohabiting, separated, divorced, widowed, or never married. Educational attainment was categorized into three levels: less than high school, high school or equivalent, and above high school. Race/ethnicity was classified as White, Mexican, Black, or other. Health conditions (cancer, diabetes, hypertension, and cerebrovascular disease) were coded as binary variables (yes/no). All covariates were derived from a combination of structured interviews, physical examinations, and laboratory assessments [11].

### 2.4 Statistical analysis

All statistical analyses accounted for the complex, multistage probability sampling design of NHANES by applying appropriate sample weights. To adjust for the combined two-year survey cycles, the primary weight variable “wtmec2yr” was divided by two. Analyses were conducted in R (version 4.3.2). The Shapiro–Wilk test was used to assess the normality of continuous variables. Normally distributed variables are presented as mean ± SD and compared across groups using one-way ANOVA. Non-normally distributed variables are expressed as medians with interquartile ranges (IQRs) and compared using the Kruskal–Wallis rank-sum test. Categorical variables are reported as frequencies (percentages) and compared using the Chi-square test. Because serum Klotho concentrations showed a skewed distribution, values were log-transformed [ln(Klotho)] to normalize the data. For subgroup analyses, Klotho concentrations were further divided into quartiles:

- Q1: 156.60–648.58 pg/mL
- Q2: 648.58–796.30 pg/mL
- Q3: 796.30–988.28 pg/mL
- Q4: 988.28–3,829.70 pg/mL

Linear regression models were employed to examine the association between ln(Klotho) and BAA. Three models were specified: Model 1 (unadjusted), Model 2 (adjusted for sex, age, and race/ethnicity), and Model 3 (fully adjusted for all covariates). Logistic regression analyses were also performed by dichotomizing BAA into positive and negative groups, with the same three levels of adjustment applied.

To evaluate whether the effect of Klotho on BAA varied by age, an interaction analysis was conducted using an ordinary least squares (OLS) regression model with an age × Klotho interaction term. In addition, potential non-linear associations between Klotho and BAA were assessed using restricted cubic spline (RCS) regression with the rms package in R, adjusting for all covariates. The fitted spline models were visualized to illustrate the dose-response relationship.

Subgroup analyses were carried out to assess associations within specific participant groups, and interaction tests were used to explore potential effect modification by demographic and health-related variables. To further characterize patterns within the data, a cluster analysis was performed using the K-prototypes algorithm, incorporating Klotho levels, BAA, and comorbidities (cancer, diabetes, hypertension, and cardiovascular disease). This approach identified three distinct participant clusters, and their characteristics were summarized to provide further insights into the interplay between Klotho, biological aging, and health status.

## 3. Results

### 3.1 Baseline information

The screening process is summarized in Fig 1. Between 1990 and 2023, data were available for 13,766 participants with serum Klotho measurements and 37,403 participants with biological age information. Data for 15 covariates were also extracted, with sample sizes ranging from 100,509–116,876 individuals. After integrating all variables, 37,403 participants remained. Following the exclusion of 31,749 participants with missing data, the final analytic sample included 5,654 individuals. The baseline characteristics of these participants are presented in Table 1. The mean biological age was 55.61 ± 0.21 years, biological age acceleration averaged 0.03 ± 0.11 years, and chronological age was 55.58 ± 0.21 years. The mean BMI was 29.24 ± 0.12 kg/m². Median serum Klotho concentration was 800.40 pg/mL (IQR: 653.90–978.00 pg/mL). Further baseline values included a median poverty index of 3.57 (IQR: 1.78–5.00), median physical activity equivalent of 2,160.00 (IQR: 720.00–5,280.00), and median serum vitamin D concentration of 3.75 (IQR: 2.05–6.30).

**Table 1:** Characteristics of the study population.

Characteristics	Total (n = 5654)	Q1 (n = 1414)	Q2 (n = 1414)	Q3 (n = 1412)	Q4 (n = 1414)	*P* value
Sex, %						0.031
Female	51.83	50.26	48.87	52.86	55.37	
Male	48.17	49.74	51.13	47.14	44.63	
Age(Year), mean(SD)	55.58(0.21)	56.59(0.33)	56.18(0.41)	55.22(0.27)	54.33(0.29)	< 0.001
BMI, mean(SD)	29.24(0.12)	29.19(0.20)	29.50(0.19)	29.09(0.23)	29.19(0.25)	0.545
Race/Ethnicity/Ethnicity, %						0.002
White	75.23	74.84	77.47	76.53	71.8	
Mexican	6.56	6.86	6.54	6.03	6.88	
Black	9.34	9.34	8.09	7.58	12.63	
Other	8.87	8.96	7.89	9.87	8.69	
Marital status, %						0.317
Married	66.76	65.32	67.59	69.13	64.96	
Living with a partner	4.25	5.11	4.08	3.48	4.45	
Separated	2.5	1.96	2.65	2.89	2.47	
Divorced	13.76	14.42	12.15	13.98	14.62	
Widowed	6.18	6.77	6.18	5.95	5.86	
Never married	6.45	6.42	7.34	4.58	7.64	
Education, %						0.781
Under high school	19.5	20.34	19.53	19.01	19.21	
High school or equivalent	24.15	24.79	22.76	25.58	23.49	
Above high school	56.28	54.88	57.71	55.42	57.3	
Poverty, median(IQR)	3.57(1.78,5.00)	3.13(1.71,5.00)	3.69(1.81,5.00)	3.77(1.85,5.00)	3.57(1.66,5.00)	0.010
Physical activity(MET/Week), median(IQR)	2160.00(720.00,5280.00)	2100.00(720.00,5520.00)	1920.00(720.00,5080.00)	2400.00(780.00,5280.00)	2040.00(720.00,5040.00)	0.625
Vitamin D(mcg), median(IQR)	3.75(2.05,6.30)	3.50(1.95,6.10)	3.85(2.05,6.35)	3.90(2.15,6.45)	3.65(2.00,6.40)	0.009
Smoking, %						0.177
Never	50.31	46.99	50.28	49.19	54.94	
Former	30.24	32.74	30.51	31.24	26.34	
Now	19.44	20.27	19.2	19.57	18.72	
Alcohol intake, %						0.010
Never	9.79	8.23	11.33	10.19	11.42	
Former	19.14	20.43	17.36	20.95	21.9	
Mild	37.73	36.45	41.37	39.59	41.26	
Moderate	14.3	16.31	15.05	14.43	14.42	
Heavy	14.11	18.58	14.89	14.85	11	
Diabetes, %						0.814
No	87.14	86.99	87.62	87.55	86.39	
Yes	12.84	13.01	12.38	12.45	13.61	
Hypertension, %						0.794
No	52.75	51.6	52.23	53.41	53.73	
Yes	47.25	48.4	47.77	46.59	46.27	
CVD, %						0.005
No	88.63	85.71	89.48	88.96	90.31	
Yes	11.37	14.29	10.52	11.04	9.69	
Cancer, %						0.364
No	87.52	88.82	86.95	86.45	88.66	
Yes	12.31	11.18	13.05	13.55	11.34	
Biological age, mean(SD)	55.61(0.21)	57.10(0.36)	56.15(0.42)	55.05(0.30)	54.16(0.33)	< 0.001
Biological age acceleration, mean(SD)	0.03(0.11)	0.50(0.22)	−0.04(0.19)	−0.17(0.14)	−0.17(0.20)	0.039

BMI, Body Mass Index; CVD, Cardiovascular Disease; IQR, Interquartile Range; SD, Standard Deviation.

Among participants, 51.83% were female, 75.23% identified as White, and 66.76% were married. Regarding education, 56.28% had a high school diploma or higher. For lifestyle factors, 50.31% reported never smoking, and 37.73% reported mild alcohol consumption. In terms of health status, 87.52% had no cancer, 87.14% had no diabetes, 52.75% reported no hypertension, and 88.63% had no cerebrovascular disease.

Comparisons across serum Klotho quartiles revealed no significant differences in BMI, physical activity, cancer status, marital status, educational level, diabetes, hypertension, or smoking status. However, significant group differences were observed for biological age, biological age acceleration, poverty index, vitamin D levels, alcohol consumption, sex, race/ethnicity, and cerebrovascular disease.

### 3.2 Association of klotho with biological age acceleration correlation analysis

To examine the association between serum Klotho levels and biological age acceleration, three linear regression models were applied, with results summarized in Table 2. Across all models, a significant negative correlation was observed between serum Klotho concentration and biological age acceleration. In detail, Model 1 yielded β = −1.06 (95% CI: −1.77 to −0.36, p = 0.005), Model 2 produced β = −1.44 (95% CI: −2.15 to −0.73, p < 0.001), and Model 3 demonstrated β = −1.30 (95% CI: −2.20 to −0.40, p = 0.01). Findings from Model 3 indicated that each 1-unit increase in the natural logarithm of Klotho (ln[Klotho]) corresponded to a mean reduction of 1.3 years in biological age.

**Table 2:** Linear regression model for ln(klotho) and biological age.

Characteristics	Model 1		Model 2		Model 3	
	β (95% CI)	*P* value	β (95% CI)	*P* value	β (95% CI)	*P* value
ln(klotho)	−1.06(−1.77,-0.36)	0.005	−1.44(−2.15,-0.73)	<0.001	−1.3(−2.20,-0.40)	0.012
Stratified by Klotho quartiles						
Q1	Ref	Ref	Ref	Ref	Ref	Ref
Q2	−0.54(−1.13, 0.05)	0.077	−0.58(−1.18, 0.01)	0.055	−0.34(−1.28, 0.60)	0.369
Q3	−0.67(−1.14,-0.21)	0.006	−0.8(−1.28,-0.31)	0.002	−0.72(−1.41,-0.04)	0.042
Q4	−0.67(−1.32,-0.02)	0.044	−0.98(−1.62,-0.34)	0.004	−0.89(−1.90, 0.12)	0.070

CI, Confidence Interval.

To further clarify this relationship, participants were stratified into quartiles based on serum Klotho concentration. In Model 1, individuals in quartiles Q3 and Q4 demonstrated significantly lower biological age acceleration compared with Q1 (Q3: β = −0.67, 95% CI: −1.14 to −0.21, p = 0.01; Q4: β = −0.67, 95% CI: −1.32 to −0.02, p = 0.04). Similarly, in Model 2, participants in Q3 and Q4 again showed reduced biological age acceleration relative to Q1 (Q3: β = −0.80, 95% CI: −1.28 to −0.31, p = 0.002; Q4: β = −0.98, 95% CI: −1.62 to −0.34, p = 0.004). In Model 3, participants in Q3 continued to show significantly lower biological age acceleration compared to Q1 (Q3: β = −0.72, 95% CI: −1.41 to −0.04, p = 0.004).

**Fig 2:**
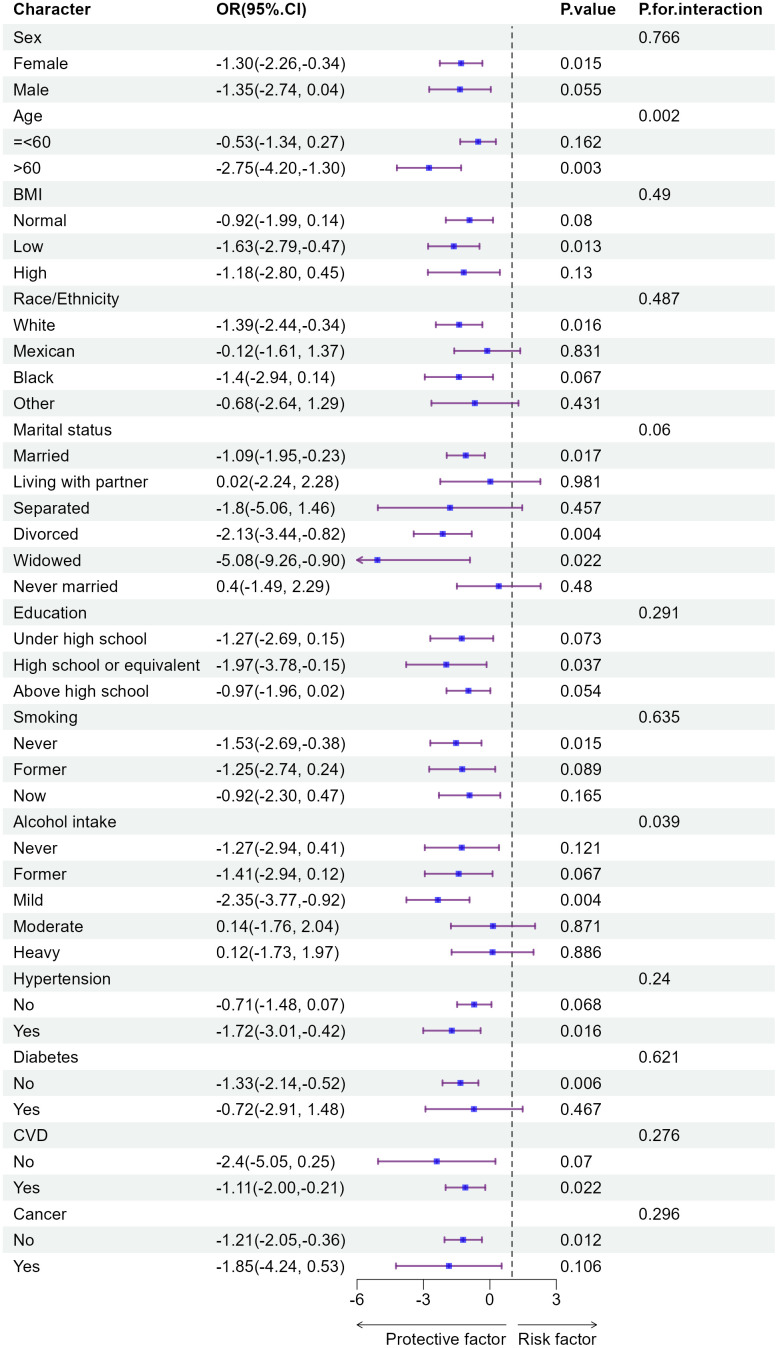
Subgroup analysis and interaction of linear regression.

The non-linear association between ln(Klotho) and biological age acceleration was further assessed using a restricted cubic spline model. A significant non-linear relationship emerged (p for nonlinearity = 0.0173), with an inflection point at 791.85 pg/mL. At this threshold, the slope approached zero, suggesting that Klotho concentrations above this level were associated with a marked decline in biological age acceleration (Fig 2).

Furthermore, binary correlation analysis was performed by categorizing biological age acceleration into two groups: positive acceleration (values > 0) and negative acceleration (values < 0). Logistic regression was then applied to evaluate the relationship between ln(Klotho) and binary biological age acceleration. In the unadjusted model, ln(Klotho) was inversely associated with biological age acceleration (OR = 0.72, 95% CI: 0.59–0.88, p = 0.002). This negative association remained robust in both the partially adjusted model (OR = 0.63, 95% CI: 0.51–0.77, p < 0.0001) and the fully adjusted model (OR = 0.62, 95% CI: 0.46–0.84, p = 0.01).

Collectively, these findings consistently demonstrate that higher levels of ln(Klotho) are linked to a reduced risk of accelerated biological aging. The logistic regression analyses reinforce a dose-response relationship, where incremental increases in ln(Klotho) correspond to progressively lower odds of experiencing biological age acceleration.

### 3.3 Subgroup analyses and interaction test

Subgroup analyses were performed to evaluate the association between ln(Klotho) and biological age acceleration across demographic and health-related categories (Fig 2). The results indicated that higher ln(Klotho) levels were consistently associated with slower biological age acceleration across several subgroups. This inverse relationship was most pronounced among females, individuals over 60 years of age, those with lower BMI, nonsmokers, moderate alcohol consumers, White participants, and those who were married, widowed, or divorced. Similarly, significant negative associations were observed among participants with a high school education or equivalent, as well as those without diabetes, hypertension, cancer, or cerebrovascular disease.

Moreover, interaction analyses revealed that age and alcohol consumption significantly modified the relationship between ln(Klotho) and biological age acceleration. Specifically, both age (p-interaction = 0.002) and alcohol use (p-interaction = 0.04) influenced the strength of this association.

**Fig 3:**
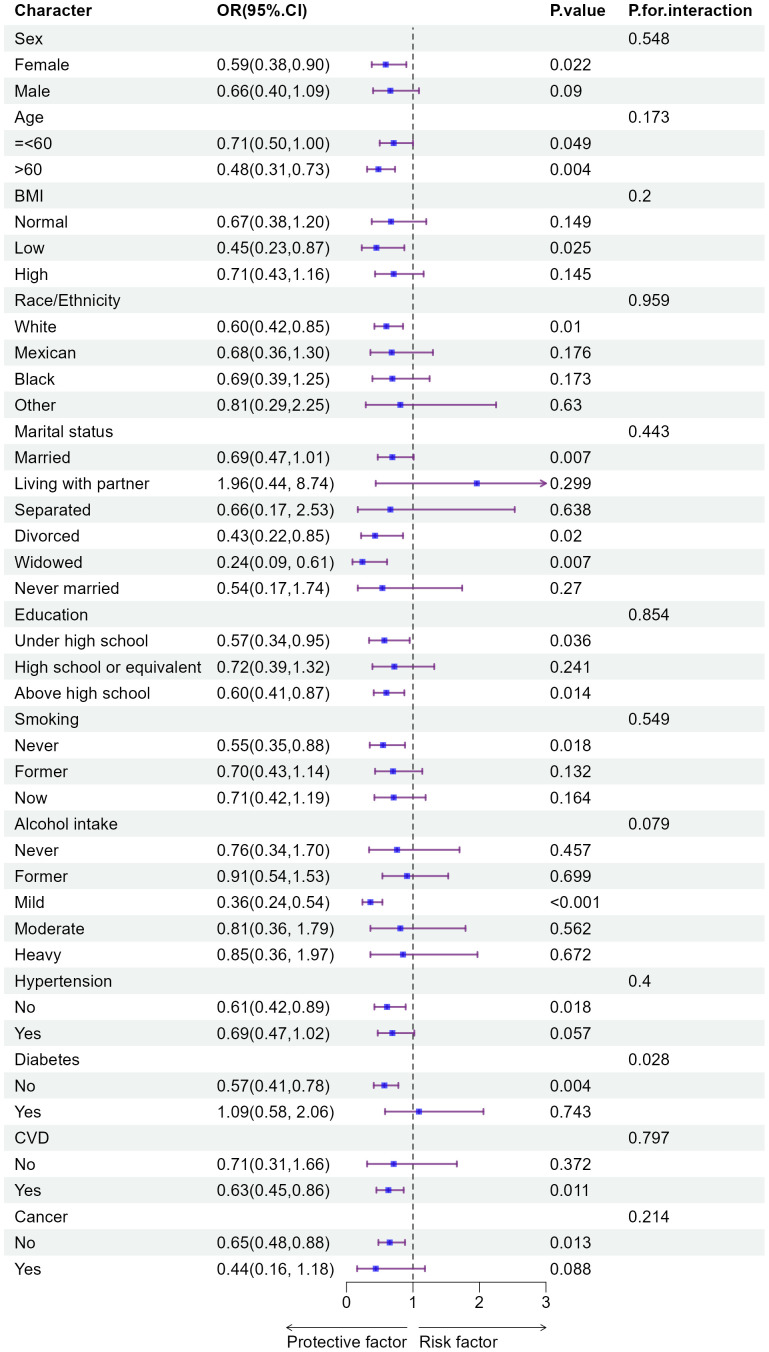
Subgroup analysis and interaction of logistic regression.

Further subgroup analyses were also conducted using the binary categorization of biological age acceleration (positive vs. negative) (Fig 3). Higher ln(Klotho) levels were linked to a reduced risk of biological age acceleration in specific groups, including females, individuals over 60 years old, those with lower BMI, never smokers, moderate drinkers, White participants, and widowed or divorced individuals. Moreover, inverse associations were also evident among those with education below or above high school equivalency, as well as those free of diabetes, hypertension, cancer, or cerebrovascular disease. Importantly, diabetes significantly modified this relationship, with a notable interaction effect (p-interaction = 0.003), suggesting that the presence of diabetes attenuates the protective association between ln(Klotho) and the risk of accelerated biological aging.

### 3.4 Klotho-age interaction

The interaction between age and ln(Klotho) levels on biological age acceleration was not statistically significant (p = 0.084), indicating that age did not significantly modify the association between ln(Klotho) and biological age acceleration in this sample.

### 3.5 Multivariable-adjusted spline analysis

**Fig 4:**
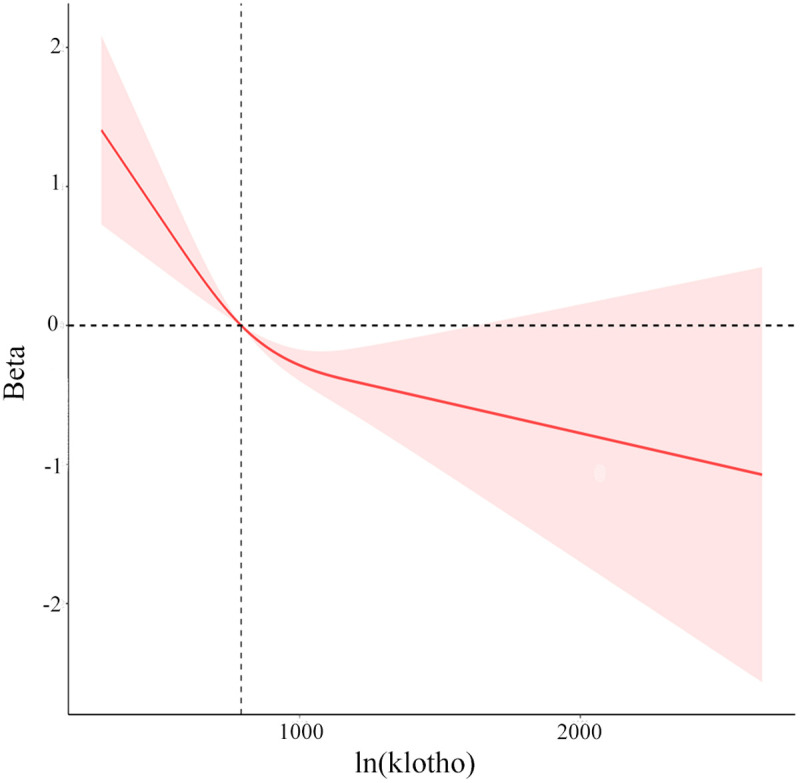
Restricted Cubic Spline (RCS) analysis of the relationship between ln(klotho) and biological age acceleration.

A restricted cubic spline model was applied to examine potential non-linear associations between Klotho levels and biological age acceleration. Klotho values were log-transformed and adjusted for all covariates, and the fitted spline curve was plotted to visualize the relationship (Fig 4). The analysis revealed a significant non-linear association (p = 0.017). The curve displayed an L-shaped pattern with an inflection point at ln(Klotho) = 791.85 pg/ml. Below this threshold, biological age acceleration declined sharply with increasing ln(Klotho), while above it, the decline was more gradual.

### 3.6 Cluster analysis

**Fig 5:**
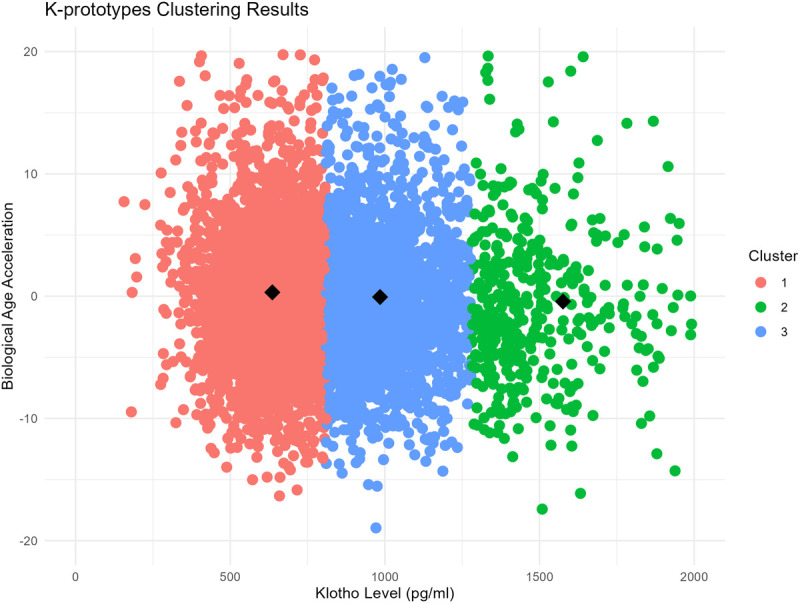
Cluster analysis of Klotho levels and biological age acceleration.

Cluster analysis revealed three distinct subgroups. Cluster 1 comprised participants with low Klotho levels (mean = 636.08 pg/ml), who showed the lowest biological age acceleration, indicating slower aging. Cluster 2 included individuals with high Klotho levels (mean = 1575.95 pg/ml), who showed slightly higher biological age acceleration, though still below the population average (Fig 5). Cluster 3, characterized by moderate Klotho levels (mean = 9984.47 pg/ml), demonstrated comparatively higher biological age acceleration than the other clusters. These findings suggest heterogeneity in aging trajectories linked to Klotho levels and comorbid conditions, identifying subgroups that may warrant deeper investigation (Fig 5).

## 4 Discussion

In this cross-sectional study, we explored the relationship between serum Klotho levels and BAA, defined as the gap between an individual’s biological and chronological age, using NHANES 2007–2010 data. These findings showed a consistent inverse association: higher serum Klotho was linked to lower BAA, suggesting that Klotho may serve a protective role against accelerated aging. Importantly, these associations remained robust across multiple regression models, indicating that Klotho captures meaningful aspects of biological aging beyond chronological time.

Klotho is a multifunctional protein that influences several processes relevant to aging, including mineral metabolism, oxidative stress, inflammation, and vascular health. Mechanistically, Klotho functions as the obligate co-receptor for FGF23, thereby regulating phosphate balance and vitamin D metabolism. Dysregulation of this axis promotes phosphate toxicity and vascular calcification, both of which accelerate vascular aging. Experimental and clinical data indicate that Klotho helps maintain vascular integrity by counteracting phosphate-induced osteogenic signaling and Wnt/β-catenin activity, processes central to arterial stiffness and calcification [12–14]. In addition to mineral metabolism, Klotho enhances resistance to oxidative stress and chronic inflammation, two major drivers of aging. It upregulates antioxidant enzymes such as superoxide dismutase, catalase, and glutathione peroxidase through Nrf2- and FOXO-mediated pathways, while also suppressing NF-κB signaling and the NLRP3 inflammasome. These actions help mitigate “inflammaging,” reduce reactive oxygen species (ROS) damage, and limit the harmful secretory phenotype of senescent cells [15,16].

Klotho is also closely connected to nutrient-sensing pathways. Preclinical studies show that it modulates insulin/IGF-1–PI3K–AKT–mTOR signaling, a pathway strongly linked to longevity. By dampening this signaling cascade, Klotho promotes stress resistance, mitochondrial stability, and autophagic balance. Evidence from animal models further suggests that Klotho can protect against insulin resistance and oxidative injury, aligning with our observation that individuals with higher Klotho had lower BAA [7,17]. Cognitive health is another domain where Klotho plays a role. Research shows that Klotho supports synaptic plasticity and dampens neuroinflammation, thereby preserving memory and cognitive performance. For example, administration of recombinant Klotho improved learning and memory in aged nonhuman primates, highlighting its translational potential. Observational studies in humans also report that higher serum Klotho is associated with better cognition in older adults [18,19]. Our regression analyses consistently confirmed these associations. Each unit increase in serum Klotho corresponded with a decrease in BAA across both unadjusted and adjusted models (Model 1: β = −1.06, p = 0.005; Model 2: β = −1.44, p < 0.001; Model 3: β = −1.30, p = 0.01). Logistic regression similarly showed that higher ln(Klotho) levels were linked to lower odds of accelerated BAA. These results are consistent with the mechanistic effects described above.

Subgroup analyses further emphasized conditions in which the Klotho–BAA link was most evident. Adults over 60 years showed stronger inverse associations, which is biologically plausible given their higher inflammatory burden and greater prevalence of chronic disease. Alcohol consumption also influenced the relationship: individuals with moderate-to-heavy alcohol intake had a stronger inverse association, consistent with studies reporting that heavy alcohol use reduces circulating Klotho levels [20]. Similarly, the inverse association was stronger in non-diabetic participants. This pattern supports the idea that intact Klotho signaling is particularly protective in metabolically healthy states, whereas diabetes-associated dysregulation may blunt its beneficial effects [21]. The RCS analysis highlighted a non-linear, L-shaped relationship, with a critical threshold around 791.85 pg/mL. Above this concentration, increases in Klotho were linked to substantial reductions in BAA, suggesting that a minimum serum level may be required to activate its anti-aging pathways fully. This finding could have practical implications for defining therapeutic targets in future interventional studies.

Collectively, these findings point to several important applications. (1). Monitoring serum Klotho could serve as a useful biomarker of biological aging, helping to identify individuals at higher risk for accelerated aging and age-related disease. However, Mendelian randomization studies have not shown a strong genetic causal link between circulating α-Klotho and lifespan, emphasizing the need for interventional data before it can be considered a therapeutic target [22]. (2). Exercise and weight reduction have been shown to raise circulating Klotho levels, with both short-term and long-term interventions linked to significant increases. These findings align with our results and suggest that lifestyle-based approaches may represent low-risk strategies to enhance Klotho and promote healthy aging [23–25]. (3). Recombinant Klotho and other pharmacological agents that enhance endogenous Klotho expression are being explored as potential interventions for aging-related cardiovascular, metabolic, renal, and neurodegenerative conditions. Findings from preclinical and early translational studies, such as improved cognition in primates, highlight the therapeutic promise [26,27].

Moreover, to move beyond correlation, future research should focus on longitudinal cohorts with repeated measures of serum Klotho and aging biomarkers to establish causal directionality. Randomized controlled trials are also needed to test both lifestyle interventions (Exercise, dietary modification, weight reduction) and pharmacological strategies (e.g., recombinant Klotho) in relation to validated biological aging markers such as epigenetic clocks or vascular stiffness indices. Mechanistic substudies could help clarify whether improvements in Klotho levels translate into measurable changes in oxidative stress, inflammation, and vascular calcification. Moreover, stratified analyses across different ages, ethnicities, and comorbidities will be necessary for understanding the contexts in which Klotho interventions may be most effective [16].

Despite its strengths, our study has several limitations. The cross-sectional design of NHANES prevents causal inference, and residual confounding from unmeasured factors cannot be excluded. The sample population primarily consisted of middle-aged and older U.S. adults, which may limit generalizability to younger or non-U.S. populations. Finally, some data were self-reported, which could introduce reporting bias. However, the consistency of our findings with mechanistic evidence supports the biological plausibility of the observed associations.

In summary, our study adds to the growing evidence that higher circulating Klotho levels are associated with slower biological aging. By integrating mechanistic insights from mineral metabolism, oxidative stress, inflammation, and cognitive resilience, these findings suggest that Klotho may serve as both a biomarker and a therapeutic target for interventions aimed at promoting healthy aging. However, definitive longitudinal and interventional studies are needed to establish causality and clinical benefit.

## 5 Conclusion

In conclusion, this study offers important insights into the intricate relationship between serum Klotho levels and biological age acceleration in middle-aged and older adults. Our results reveal a strong inverse association, showing that higher circulating Klotho concentrations are linked to slower biological age acceleration. These findings suggest that Klotho may exert a protective role against the aging process, particularly among older individuals, moderate to heavy alcohol consumers, and those without diabetes. The observation of a non-linear, L-shaped dose–response relationship further underscores the possibility of a threshold effect, whereby maintaining serum Klotho levels above 791.85 pg/ml could meaningfully reduce the risk of accelerated biological aging. Collectively, these results support the utility of Klotho as a potential biomarker of biological aging and highlight its promise as a therapeutic target for strategies aimed at promoting healthy aging and reducing susceptibility to age-related disorders.

Furthermore, future studies should prioritize longitudinal research to establish causal relationships and to clarify the biological pathways through which Klotho modulates aging. Moreover, the development and evaluation of interventions designed to elevate serum Klotho levels may represent a promising avenue for extending health span and improving quality of life in aging populations. Advancing our understanding of Klotho’s role in the biology of aging could ultimately inform innovative approaches to address the growing global health challenges posed by demographic aging.
